# MicroRNA-322 inhibits inflammatory cytokine expression and promotes cell proliferation in LPS-stimulated murine macrophages by targeting NF-κB1 (p50)

**DOI:** 10.1042/BSR20160239

**Published:** 2017-01-17

**Authors:** Kai Zhang, Fengling Song, Xiaoxia Lu, Wenxun Chen, Chunxiao Huang, Lexing Li, Danyang Liang, Shengbo Cao, Hanchuan Dai

**Affiliations:** College of Veterinary Medicine, Huazhong Agricultural University, Wuhan, Hubei 430070, China

**Keywords:** miR-322, NF-κB1 (P50), Inflammation

## Abstract

Inflammation is the body’s normal self-protection mechanism to eliminate pathogens and resist pathogen invasion. The excessive inflammatory response may lead to inflammatory lesions. The mechanisms accounting for inflammation remain hazy. miRNAs have been proposed to have crucial effects on inflammation. In the present study, we reported that lipopolysaccharide (LPS)-stimulation increased the expression levels of inflammatory cytokines and the cell-cycle progression was suppressed in RAW264.7 cells. Meanwhile, the expression of *miR-322* was significantly down-regulated after LPS treatment. Bioinformatics predictions revealed a potential binding site of *miR-322 * in 3′-UTR of NF-κB1 (p50) and it was further confirmed by luciferase assay. Moreover, both the mRNA and protein levels of NF-κB1 (p50) were down-regulated by *miR-322* in RAW264.7 cells. Subsequently, we demonstrated that *miR-322* mimics decrease in the expression levels of inflammatory cytokines and cell-cycle repression can be rescued following LPS treatment in RAW264.7 cells. The anti-inflammatory cytokines expression including IL-4 and IL-10 were significantly up-regulated. Furthermore, *miR-322* could also promote RAW264.7 cells proliferation. These results demonstrate that *miR-322* is a negative regulator of inflammatory response by targeting NF-κB1 (p50).

## Introduction

Inflammation response plays a crucial role in eliminating pathogens and resisting pathogen invasion [1]. The excessive inflammatory response may lead to inflammatory lesions. Increasing evidence suggests that inflammatory response is related to many diseases and cancer [2]. The complex process of inflammatory response is initiated by innate immune system receptors, which recognize a lot of pathogens or damage signals and precisely regulate immune response. Cells expressing pathogen-recognition receptors (PRRs) are able to present pathogens, antigens and secrete cytokines and chemokines [3,4]. PRRs, such as toll-like receptors (TLRs), activate cellular signalling pathways that result in NF-κB translocating into nucleus, and lead to the production of inflammatory cytokines and chemokines, which directly contribute to inflammatory lesions and immune diseases [5].

NF-κB is an important transcription factor that regulates the transcription and expression of multiple genes. It is closely related to the cell activation, cell proliferation, immune response and inflammatory reaction process [6]. NF-κB comprises a family of proteins such as RelA (p65), RelB, c-Rel, NF-κB1 (p105/p50) and NF-κB2 (p100/p52). The protein p50, the transcript of NF-κB1, is an important subunit of NF-κB. It regulates gene transcription through combination with p65, which is another important subunit of NF-κB. When the signalling pathway is activated, p50/p65 exposes nuclear localization signal and translocates into the nucleus, regulating the transcription of downstream genes. Therefore, p50 protein levels are critical for activation of NF-κB signalling pathway and body’s inflammatory reaction [5].

miRNAs are naturally occurring non-coding small RNA molecules capable of degrading the target mRNA or repressing its translation by targeting mRNA 3′-UTR region [7,8]. Overwhelming evidence shows that miRNA-mediated post-transcriptional regulation plays a critical role in inflammatory response and immune regulation [9,10]. *miR-146* is probably one of the most studied miRNA that regulates inflammatory response by targeting TRAF6 and IRAK1 following lipopolysaccharide (LPS)-stimulation [11]. *miR-125b* can target TNF-α resulting in inhibition of inflammatory response [12]. *miR-181a* and *miR-155* regulate inflammation responses by targeting IL-1α respecting TAB2 and SOCS1 [13,14]. However, it remains largely unknown as to how inflammation is regulated by miRNA in immune response.

*miR-322* is the homologue of human *miR-424*, which is differentially expressed in a variety of disease conditions and is highly conserved in different cells [15]. *miR-322/424* is a member of *miR-15/107* family, also known as *miR-16* family [16,17]. It is involved in the regulation of cell proliferation, cell differentiation, diabetes and male infertility [15,18–20]. In our preliminary study, *miR-322* was predicted to target several sites of inflammatory factors using the software programs. Little is known about the involvement of *miR-322* during inflammatory response. RAW264.7 was a mouse peritoneal macrophage cell line established from a tumour induced by Abelson murine leukaemia virus. It is one of the commonly used inflammatory cell models. Here, we found that the level of *miR-322* was down-regulated in RAW264.7 cells by administration of LPS. We also showed that *miR-322* mimic transfection resulted in an inhibition in pro-inflammatory cytokines mRNA expression, such as IL-1β, IL-6, TNF-α and increased anti-inflammatory cytokines IL-4 and IL-10 expression. Besides, NF-κB1 (p50) was identified as a functional target, through which *miR-322* acted as a negative regulator in macrophage inflammatory response. Moreover, *miR-322* may promote cell-cycle procession and cell proliferation. Our findings demonstrate that the level of *miR-322* is down-regulated by LPS-stimulation and *miR-322* is a negative regulator of the immune response.

## Materials and methods

### RAW264.7 cells’ culture and treatment

RAW264.7 was a mouse peritoneal macrophage cell line established from a tumour induced by Abelson murine leukaemia virus. It is one of the commonly used inflammatory cell models. Cells were cultured in DMEM (Hyclone) medium supplemented with 10% FBS at 37°C in 5% CO_2_. RAW264.7 cells were seeded in six-well plates at a density of 2 × 10^5^ cells/well. Twenty four hours later, the cell medium was replaced with fresh medium. Cells were collected at 0, 2, 4, 8, 12 and 24 h after 1 μg/ml LPS (Sigma–Aldrich, U.S.A.) induction.

### *miR-322* mimics transfection

*miR-322* mimics and *miR-322* inhibitors were purchased from GenePharma (China). RAW264.7 cells were seeded into six-well plates for 12 h. The cells were replaced with fresh medium (DMEM + 10% FBS) and transfected with 50 nM *miR-322* mimics and *miR-322* inhibitors using Lipofectaime 2000 (Invitrogen TM, U.S.A.) according to the manufacturer’s instructions. After transfection for 24 h, the medium was replaced with fresh medium containing 1 µg/ml LPS. The cells were collected after LPS induction for 8 h.

### Quantitative real-time PCR 

Total RNA was extracted from treated cells with TRIzol (Invitrogen) according to the instructions of the manufacturer. For mRNA analysis, reverse transcription was performed using a first-strand cDNA synthesis kit (Toyobo, Japan). To quantify mature *miR-322* expression, a commercial Bulge-Loop™ miRNA quantitative reverse transcription detection method was used with *miR-322-*specific RT primer (Table 1). All gene transcripts were measured by quantitative real-time PCR (qPCR) using a 7500 real-time PCR system (Applied Biosystems) and SYBR Green PCR master mix (Toyobo, Japan). qPCR primers of *miR-322* and the endogenous control gene *U6* were from RiboBio (China), whereas other primers were designed by the Primer Express software and synthesized from Invitrogen (Table 2). Fold change was calculated using the 2^−ΔΔ*C*^_^t^_ method of relative quantification. All experiments were conducted in triplicate.

**Table 1:** Nucleic acid sequences for miRNAs

Gene names	Sequence (5′ to 3′)
*miR-322* inhibitors	UUCAAAACAUGAAUUGCUGCUG
Inhibitors NC	CAGUACUUUUGUGUAGUACAA
*miR-322* mimics	Sense: CAGCAGCAAUUCAUGUUUUGAA
	Antisense: CAAAACAUGAAUUGCUGCUGUU
Mimics NC	Sense: UUCUCCGAACGUGUCACGUTT
	Antisense: ACGUGACACGUUCGGAGAATT
*miR-322* stem loop-primer	GTCGTATCCAGTGCAGGGTCCGAGGTATTCGCACTGG
	ATACGACTTCAAA
*miR-322*	Sense: CAGCAGCAATTCATGTTTTGAA
	Antisense: GTGCAGGGTCCGAGGT
*U6*	Sense: CTCGCTTCGGCAGCACA
	Antisense: AACGCTTCACGAATTTGCGT

**Table 2:** Primers for mRNA

Primer	Sequence 5′→3′
NF-κB1 UTR-3′-WT	Sense: CCCTCGAGTTCCAACACCGCATAAACCAAAGC
	Antisense: ATTTGCGGCCGCCTGAATCCTTAACTGCTAGGC
NF-κB1 UTR-3′-MUT	Sense: GTTGTTATTGTGATGGTCCCTCTG
	Antisense: CAGAGGGACCATCACAATAACAAC
GAPDH	Sense: GGTGAAGGTCGGTGTGAACG
	Antisense: CTCGCTCCTGGAAGATGGTG
IL-1β	Sense: CCTAAAGTATGGGCTGGACTG
	Antisense: CTCGCTCCTGGAAGATGGTG
IL-6	Sense: CGGAGAGGAGACTTCACAGAG
	Antisense: ATTTCCACGATTTCCCAGAG
TNF-α	Sense: TGAAGGGAATGGGTGTTCAT
	Antisense: TTGGACCCTGAGCCATAATC
IL-4	Sense: GGTCTCAACCCCCAGCTAGT
	Antisense: GCCGATGATCTCTCTCAAGTGAT
IL-10	Sense: CGGGAAGACAATAACTG
	Antisense: CATTTCCGATAAGGCTTGG
Cyclin D1	Sense: TGACACCAATCTCCTCAACG
	Antisense: CTCACAGACCTCCAGCATCC
Cyclin E	Sense: CACCTCCAGAACACCACTGA
	Antisense: AACCTACAACACCCGAGCAG
P21	Sense: CAAAGTGTGCCGTTGTCTCT
	Antisense: TCAAAGTTCCACCGTTCTCG
P27	Sense: GCAGATACGAGTGGCAGGA
	Antisense: ACGAGTCAGGCATTTGGTC

### Computational prediction of the miRNA targets

To further analyse the functions of *miR*-322, we used three computational approaches, TargetScan (http://www.targetscan.org/), PicTar (http://pictar.mdc-berlin.de/) and miRBD (http://mirdb.org/miRDB/), to predict the targets of *miR-322* in the TLR signalling pathways. Then, the miRNA-binding sites in target genes and the binding free energy were analysed and calculated on the website (http://bibiserv.techfak.uni-bielefeld.de/rnahybrid/) [21].

### Luciferase reporter assays

293T cells were cultured in DMEM medium and seeded in six-well plates at a density of 2 × 10^5^ cells/well. The 3′-UTRs of mouse NF-κB1 (p50) and their corresponding mutated 3′-UTRs were amplified by PCR using the primers shown in Table 1 and cloned into psiCheck-2 dual-luciferase reporter vector (Promega). Co-transfection was performed with constructed plasmid, miRNA mimics or inhibitors using Lipofectamine 2000. *Renilla* luciferase activities were measured using the Dual-Luciferase Reporter Assay System (Promega) according to the manufacturer’s instructions.

### Western blotting

Protein concentrations were extracted from cells and measured with the BCA Protein Assay kit (Thermo Scientific), then subjected to SDS/PAGE (10%) gel and transferred to PVDF membranes. The membranes were blocked with 5% BSA. Protein was blotted with different antibodies following the published protocol. All antibodies were obtained from Cell Signaling Technology (U.S.A.). The results were used to visualize the proteins by the ECL reagents (Thermo Scientific) and quantified using Image J 1.44 software (National Institute of Health, Bethesda, Maryland) after densitometric scanning of the films.

### Flow cytometry

RAW264.7 cells were transfected with *miR-322* mimics or negative control for 24 h and stimulated with LPS for 24 h. Cells were harvested, washed twice in PBS and fixed in 75% ethanol at 4°C overnight. Staining for DNA content was performed with 50 mg/ml propidium iodide (Sigma, U.S.A.) and 1 mg/ml RNase A for 30 min. Analysis was performed on FACS flow cytometer (BD Co., U.S.A.). The percentage of cells in G_0_/G_1_-, S- and G_2_/M-phases was determined using the Modfit LT Cycle Analysis software.

### MTT assay

RAW264.7 cells (2 × 10^5^ per well) were seeded in 96-well plates in a final volume of 100 μl. After growing to 70% confluence, 50 nM *miR-322* mimics, inhibitors or negative controls were transfected by lipofectamine 2000. After 24 h, the cells were stimulated with LPS at a dose-gradient of 0, 0.5, 1.0, 1.5 and 2.0 μg/ml. The MTT assay was performed according to the manufacturer’s instructions.

### Statistical analysis

Each result represents the mean ± S.E.M. in at least three experiments with similar results. Analyses were conducted using paired two-tailed Student’s *t* test with Prism 5.0 (GraphPad Software, San Diego, California) or one-way ANOVA with SPSS 20.0.

## Results

### LPS stimulates the inflammatory cytokines expression and inhibits the cell-cycle progression in RAW264.7 cells

**Figure 1:**
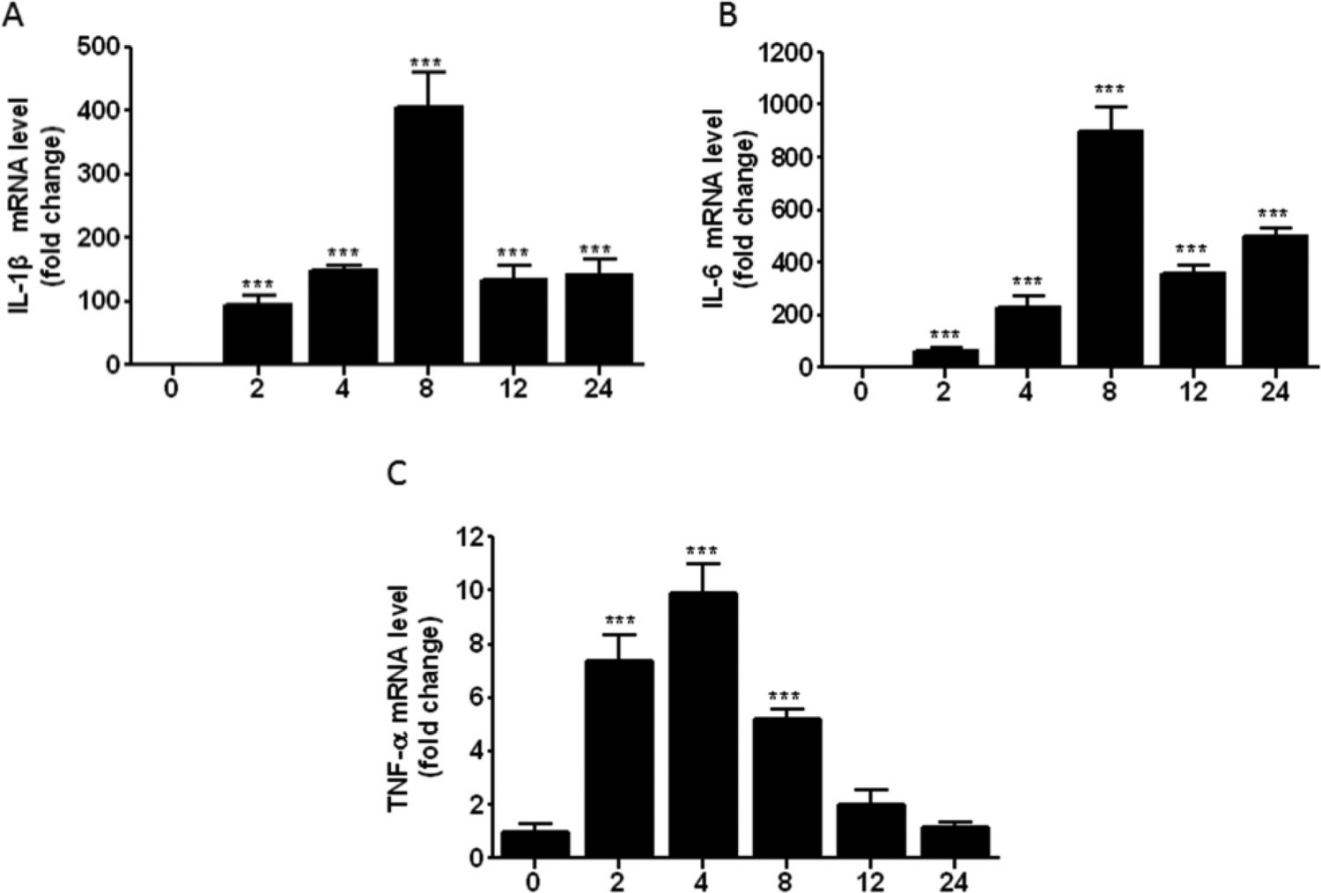
LPS increases the mRNA expression of inflammatory cytokines in RAW264.7 cells RAW264.7 cells were incubated with 1 μg/ml LPS for 0, 2, 4, 8, 12 and 24 h. Total RNA was extracted and the mRNA expression of IL-1β (**A**), IL-6 (**B**) and TNF-α (**C**) were detected with qPCR. Results are representative of three independent experiments performed in triplicate (***P<0.001).

**Figure 2:**
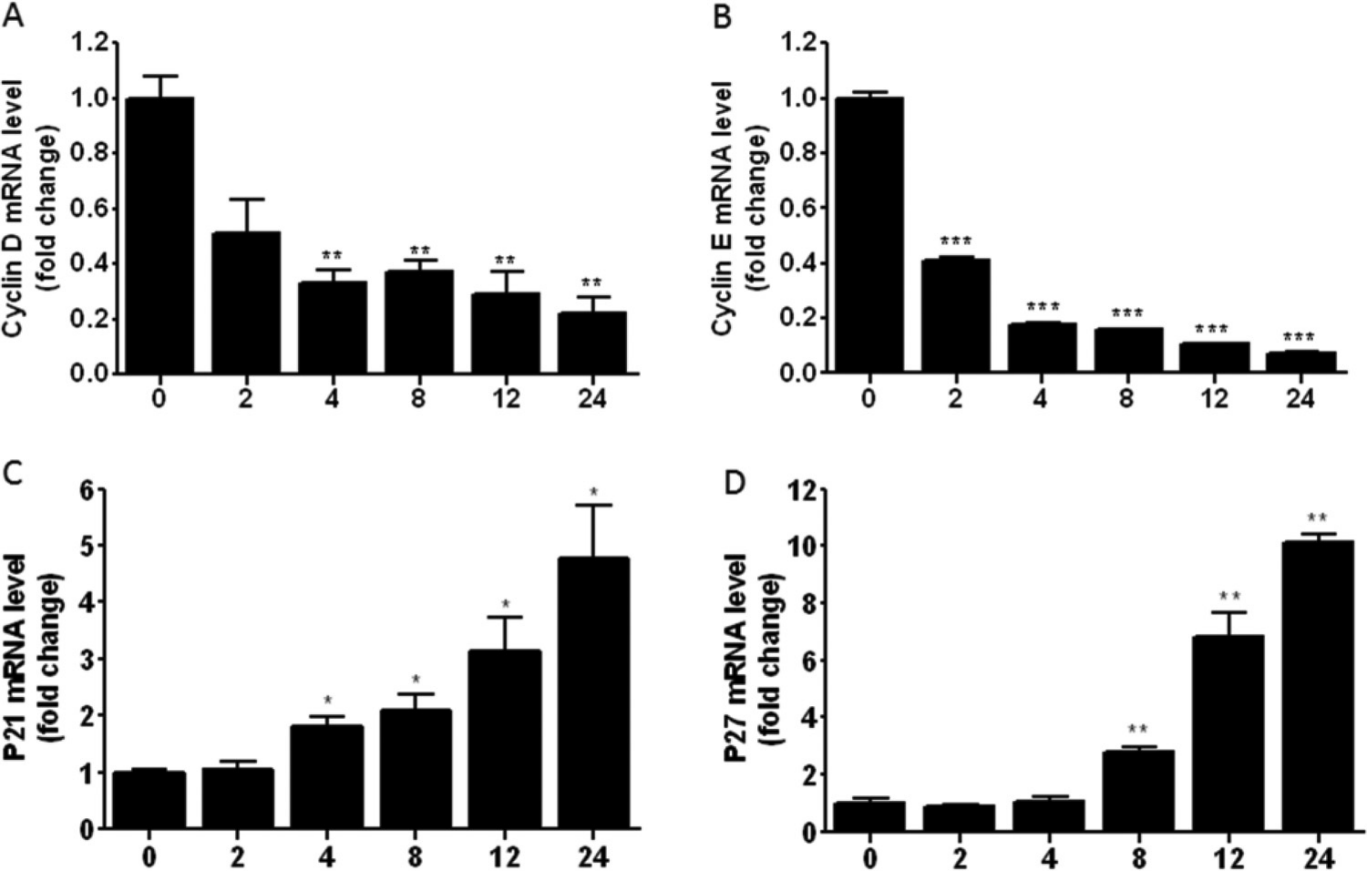
LPS inhibits the process of cell cycle of RAW264.7 cells RAW264.7 cells were incubated with 1 μg/ml LPS for 0, 2, 4, 8, 12 and 24 h. Total RNA was extracted and the mRNA expressions of cyclin D (**A**), cyclin E (**B**), P21 (**C**) and P27 (**D**) were detected with qPCR. Results are representative of three independent experiments performed in triplicate (*P<0.1, **P<0.01 and ***P<0.001).

LPS-stimulation could activate TLR4/NF-κB signalling pathways and lead to inflammatory response, which could contribute to the cell-cycle procession [5]. The excessive inflammatory response may lead to cell lesions and affect cell proliferation, differentiation and other physiological states. To investigate the effect of LPS on inflammatory response and cell cycle, we detected the mRNA expression levels of inflammatory cytokines, cell-cycle gene in LPS-induced RAW264.7 cells. The results revealed that LPS could promote the mRNA expression level of IL-1β, IL-6 and TNF-α. The expression level of inflammatory cytokines was highest at 4 h or 8h opon with 1 μg/ml LPS,then the expression level decreased gradually (Figure 1). The mRNA expressions of cyclin D and cyclin E were significantly increased, and P21 and P27 were significantly suppressed with time-dependent manner. The results indicated that LPS could mediate inflammatory response and inhibit the procession of cell cycle (Figure 2).

### *miR-322* is down-regulated in LPS-treated RAW264.7 cells

**Figure 3:**
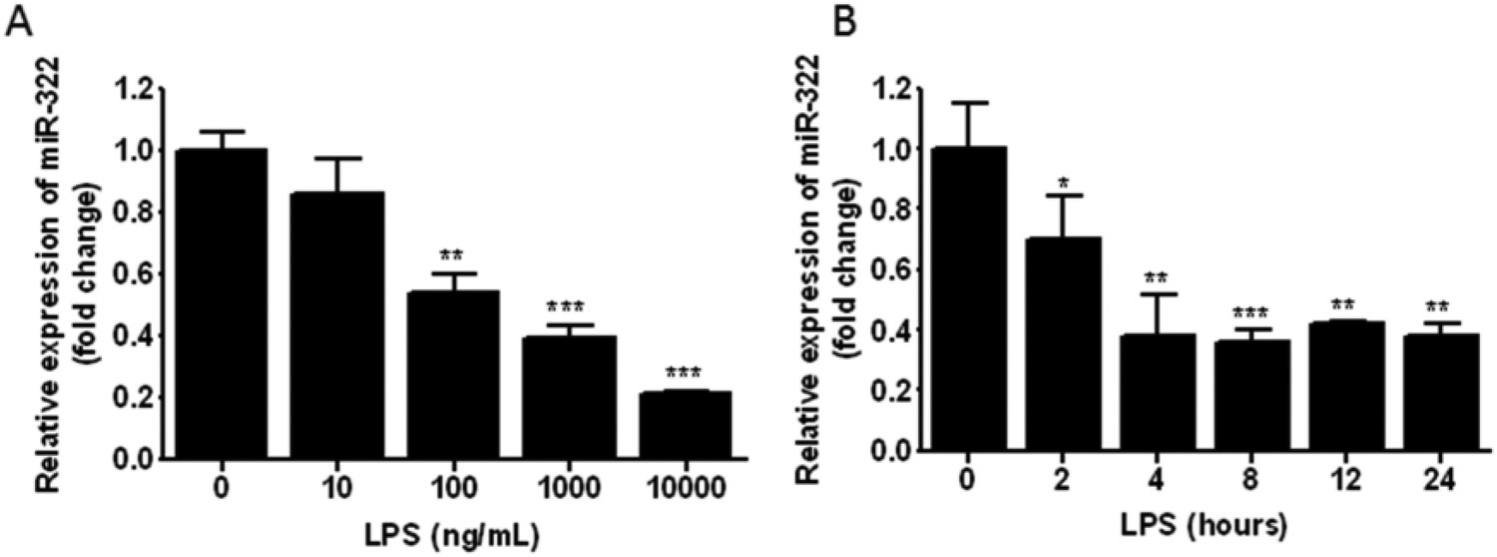
Expression of miR-322 is down-regulated in LPS-treated RAW264.7 cells (**A**) RAW264.7 cells were incubated with different doses of LPS for 24 h. Total RNA was extracted and *miR-322* levels were detected with qPCR and normalized to endogenous U6. (**B**) RAW264.7 cells were treated with 1 μg/ml LPS at indicated times. *miR-322* levels were detected with qPCR and normalized to endogenous U6. Results are representative of three independent experiments performed in triplicate (*P<0.1, **P<0.01 and ***P<0.001; the marked group was compared with the group without LPS treatment).

miR-*322* plays an important role in cell proliferation, cell differentiation, diabetes and male infertility [18–20]. The role in inflammatory response is not exactly reported. To explore the function of *miR-322* in LPS-induced inflammatory response, the expression level of *miR-322* in LPS-treated RAW 264.7 cells was tested. *miR-322* expression level was dramatically suppressed upon LPS-stimulation and showed a time-dependent manner (Figure 3A). Moreover, the decrease in *miR-322* exhibited a dose-dependent manner with LPS stimulation (Figure 3B). These results suggest that LPS could down-regulate *miR-322* expression. *miR-322* may be involved in the regulation of LPS-mediated inflammatory response.

### *miR-322* inhibits LPS-stimulated inflammatory cytokine mRNA expression

**Figure 4:**
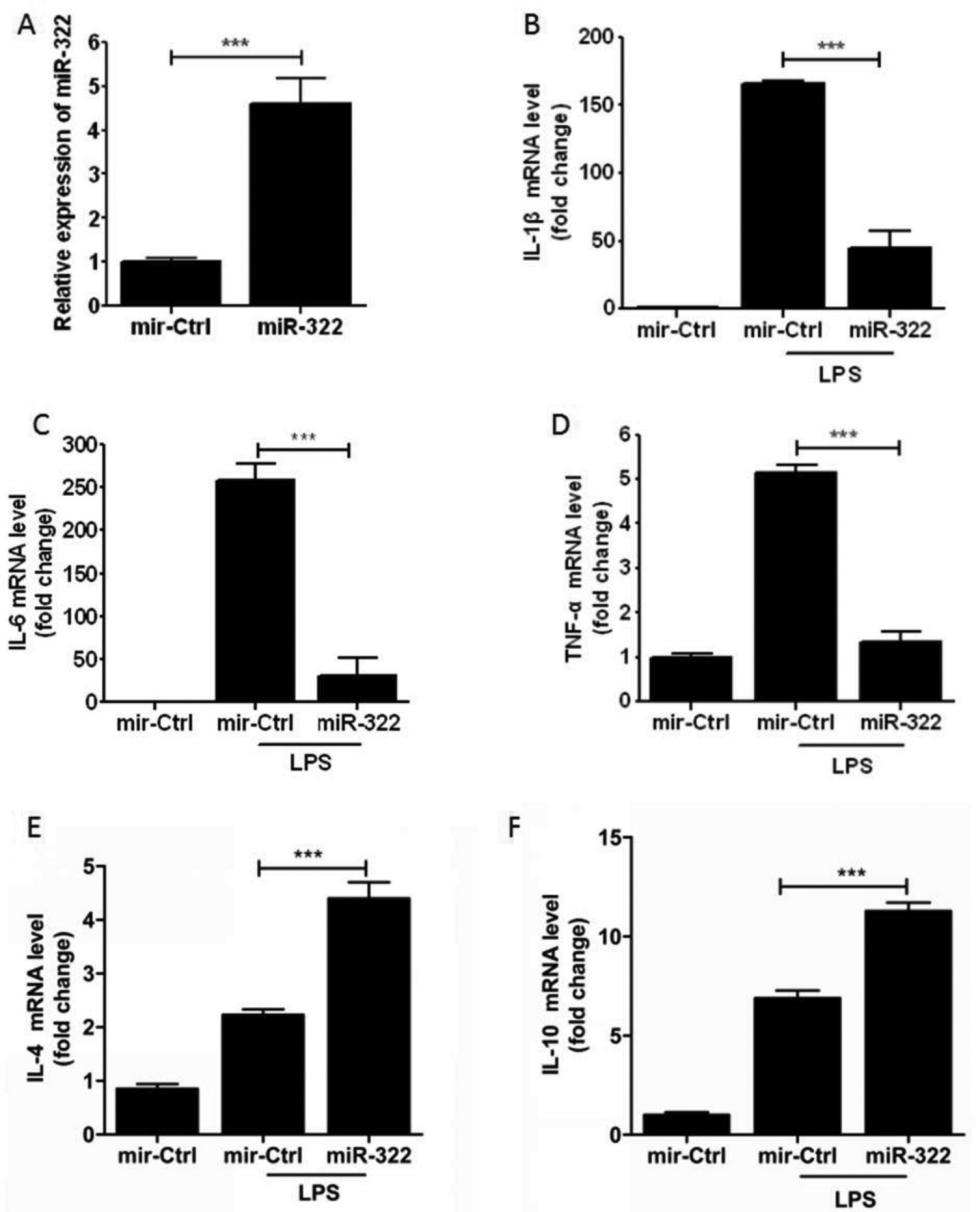
*miR-322* inhibited macrophage inflammatory response RAW264.7 cells were transfected with *miR-322* mimics or the negative control for 24 h and then incubated with LPS (1 μg/ml) for 8 h. (**A**) *miR-322* was measured by qPCR and normalized to endogenous U6. (**B**–**D**) mRNA levels of IL-1β, IL-6 and TNF-α were detected with qPCR. (**E** and **F**) mRNA levels of IL-4 and IL-10 were detected with qPCR. Results are representative of three independent experiments performed in triplicate (***P<0.001).

To assess the involvement of *miR-322* on inflammatory cytokine expression, *miR-322* was overexpressed in RAW264.7 cells by transfection of *miR-322* mimics. Cells were treated with 1 μg/ml LPS for 8 h. *miR-322* levels and the mRNA expression levels of inflammatory cytokines including IL-1β, IL-6, TNF-α and anti-inflammatory cytokines including IL-4 and IL-10 were measured by qPCR. We found that the expression of *miR-322* increased after transfection of* miR-322* mimics (Figure 4A). Inflammatory cytokines ( IL-1β, IL-6 and TNF-α) were suppressed after LPS exposure compared with control while overexpression of* miR-322* (Figure 4B–4D) and anti-inflammatory (IL-4 and IL-10 ) were significantly up-regulated (Figure 4E and 4F). These data demonstrated that *miR-322* may negatively regulate LPS-stimulated inflammatory cytokine expression.

### *miR-322* rescues cell-cycle procession and promotes cell proliferation in RAW264.7 cells

**Figure 5:**
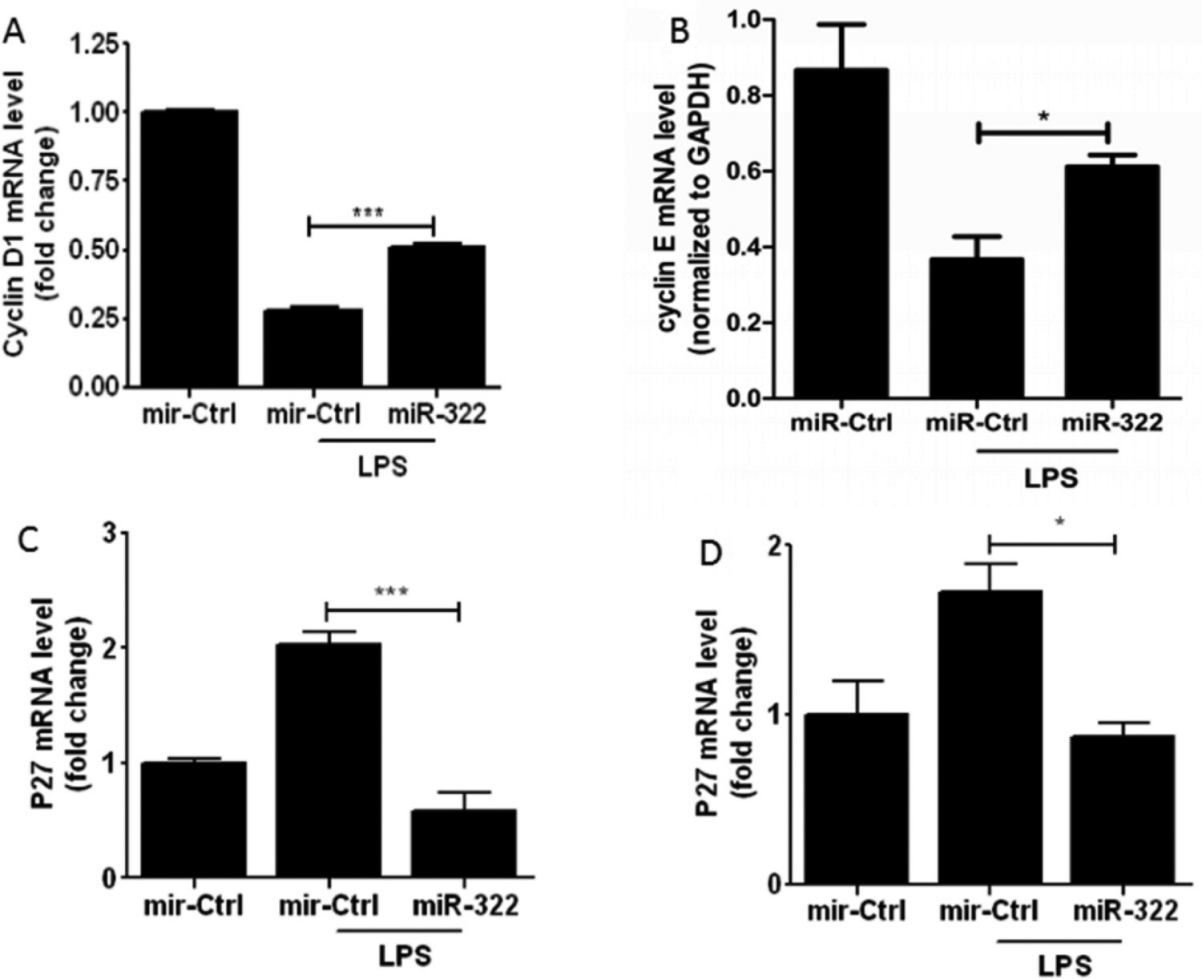
*miR-322* promotes cell cycle process of RAW264.7 cells RAW264.7 cells were transfected with *miR-322* mimics or the negative control for 24 h and then incubated with or without LPS (1 μg/ml) for another 24 h. *Cyclin D*, *cyclin E*, *P21* and *P27* mRNA levels were detected with qPCR. Results are representative of three independent experiments performed in triplicate (*P<0.1 and ***P<0.001).

**Figure 6:**
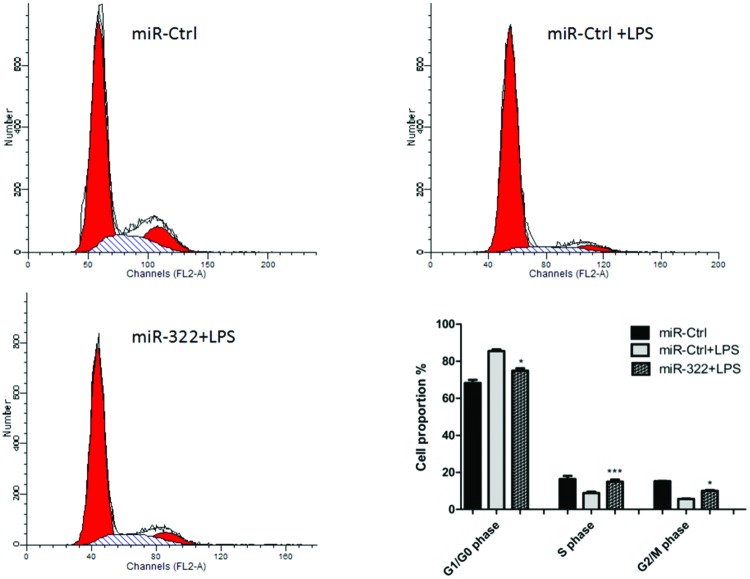
*miR-322* rescues cell-cycle procession in RAW264.7 cells RAW264.7 cells were transfected with *miR-322* mimics for 24 h, cell-cycle analysis was performed at 24 h after LPS-stimulation. The diagram above displays the percentage changes in G_0_/G_1_ and G_2_/M.

To investigate the involvement of *miR-322* on cell-cycle procession in RAW264.7 cells, *miR-322* was overexpressed in RAW264.7 cells for 24 h following LPS-stimulation. Then, cell cycle-related genes *cyclin D*, *cyclin E*, *P21* and*P27 *mRNA levels were detected by qPCR. The results showed that mRNA expressions of cyclin D and cyclin E were significantly increased, whereas those of P21 and P27 were significantly suppressed (Figure 5). These findings indicated that *miR-322* appeared to promote cell-cycle procession by regulating expression of cell cycle-related genes. To further confirm the impact of *miR-322* on cell-cycle procession, flow cytometry was applied. Cell cycle was arrested in G_0_/G_1_ in RAW264.7 cells following LPS-stimulation. Transfection of *miR-322* increased S and G_2_/M and decreased the population of cells in G_0_/G_1_ (Figure 6). It indicated that LPS-induced cell-cycle repression can be rescued by *miR-322*.

**Figure 7:**
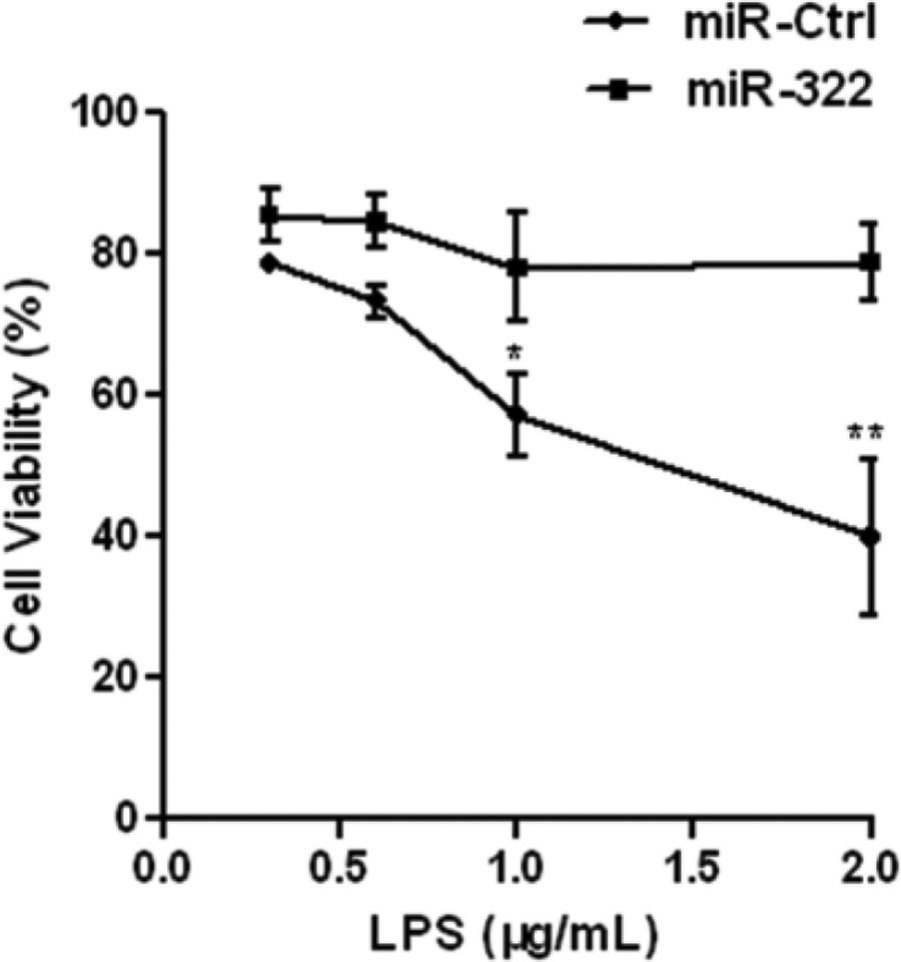
*miR-322* promotes RAW264.7 cells proliferation RAW264.7 cells were transfected with *miR-322* mimics or negative control for 24 h and then incubated with LPS at a dose gradient of 0, 0.5, 1.0, 1.5 and 2.0 μg/ml. After 24-h incubation, the MTT assay was performed to determine the proliferation of RAW264.7 cells. Results are representative of three independent experiments performed in triplicate (*P<0.1 and **P<0.01).

As inflammatory response may lead to cell lesions and affect cell proliferation, we wished to further elucidate the effect of *miR-322* on RAW264.7 cells proliferation. *miR-322* mimics were transfected to RAW264.7 cells and cells were treated with LPS at a dose gradient of 0, 0.5, 1.0, 1.5 and 2.0 μg/ml for 24 h. MTT assay demonstrated that the cell viability was significantly reduced with a dose-dependent manner. *miR-322* could promote RAW264.7 cells proliferation (Figure 7). All these findings suggested that *miR-322* could promote procession of cell cycle and cell proliferation.

### *miR-322* directly targeted NF-κB1 (p50) through 3′-UTR interaction

**Figure 8:**
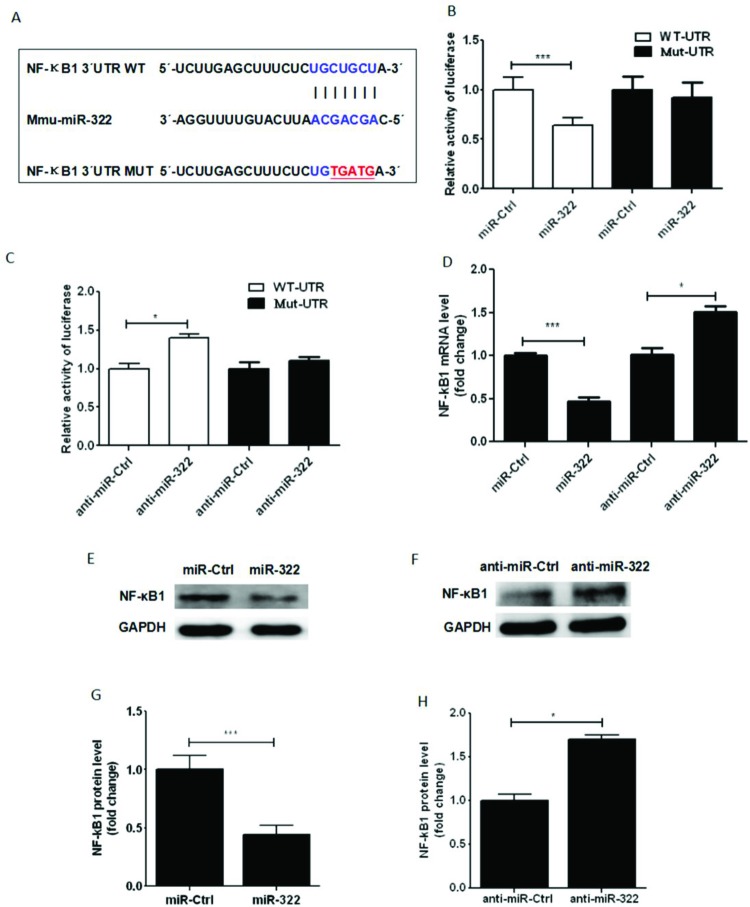
*miR-322* down-regulated NF-κB1 (p50) expression through direct 3′-UTR interaction in RAW264.7 cells Schematic diagram of *miR-322*-binding site in NF-κB1 3′-UTR region by bioinformatics analyses. Mutant NF-κB1 (p50) 3′-UTR included several mutations in *miR-322*-binding sites. The blue indicates the seed region and the red indicates the mutant sites (**A**). RAW264.7 cells were cotransfected with psiChecK-NF-κB1 (p50) 3′-UTR (WT–3′-UTR) or psiChecK-NF-κB1 (p50) 3′-UTR mutant (Mut-3′-UTR) along with *miR-322* mimics or mimics control, anti-*miR-322* or anti-*miR-322* control. After 24 h, the luciferase activity was determined and normalized to *Renilla* luciferase activity of three independent experiments (**B** and **C**). RAW264.7 cells were transfected with *miR-322* mimics or mimics control, anti-*miR-322* or anti-*miR-322* control for 24 h, and then NF-κB1 (p50) mRNA levels were measured with qPCR and normalized to GAPDH (**D**). NF-κB1 (p50) and GAPDH were detected by Western blotting at 48 h after RAW264.7 cells were transfected with *miR-322* mimics or mimics control, anti-*miR-322* or anti-*miR-322* control respectively (**E** and **F**). The NF-κB1 (p50) protein expression levels are expressed as a percentage of the *miR-322* group in its control or anti-*miR-322* group in its control (**G** and** H**). Results are representative of three independent experiments performed in triplicate (*P<0.1 and ***P<0.001).

To further elucidate the mechanisms of *miR-322* regulating LPS-induced inflammatory response, Targetscan and miRanda were used to predict *miR-322* targets that are related to NF-κB signalling. The analysis results revealed that an *miR-322* seed sequence was predicted in the NF-κB1 3′-UTR. NF-κB1, also named p50, is an important subunit of NF-κB, which affects inflammatory reaction seriously. To test whether *miR-322* can directly bind to NF-κB1 (p50) 3′-UTR, we generated a luciferase construct containing the NF-κB1 (p50) 3′-UTR with the predictive *miR-322*-binding site. In addition, a luciferase construct containing the NF-κB1 (p50) 3′-UTR with a mutation at the putative *miR-322* seed sequence was generated as the control construct (Figure 8A). Two hundred and ninety three T-cells were cotransfected with a psiChecK-2 vector, the luciferase-wild-type NF-κB1 (p50)-3′-UTR (WT-3′-UTR) or the luciferase mutant (MUT-3′-UTR) report vector, as well as *miR-322* mimics or inhibitors. *miR-322* reduced the WT-3′-UTR but not MUT-3′-UTR luciferase levels. These results indicated that *miR-322* could directly target NF-κB1 (p50)-3′-UTR (Figure 8B and 8C). In order to further confirm the regulatory function of *miR-322* to NF-κB1 (p50), we used qPCR and Western blotting to detect mRNA and protein levels of NF-κB1 (p50) in cells transfected with *miR-322* mimics or inhibitors. NF-κB1 (p50) mRNA and protein levels were significantly decreased in cells transfected with *miR-322* mimics, not with *miR-322* inhibitors (Figure 8D–8G). These data suggested that *miR-322* repressed NF-κB1 (p50) expression by directly targeting 3′-UTR of NF-κB1 (p50) mRNA.

## Discussion

Inflammation is the normal self-protection mechanism to eliminate pathogens and resist pathogen invasion [22,23]. The excessive inflammatory response and prolonged inflammation may lead to cell lesions or tissue damage [24]. Here, we found that *miR-322* expression is markedly suppressed in LPS-treated RAW264.7 cells. Subsequent finding suggested that *miR-322* targets 3′-UTR of NF-κB1 (p50) and negatively regulates LPS-modulated inflammatory cytokine expression.

*miR-322*, a member of an evolutionarily conserved *miR-16* family, contributes to promoting osteoblast and muscle cells proliferation, differentiation by directly targeting Tob2 and Cdc25A [18,25]. *miR-322* also plays a crucial role in diabetes, male infertility and other biological processes [19,20]. However, until now, the role of *miR-322* in inflammatory response remains to be further studied. Emerging results indicated that LPS activates NF-κB signalling pathways, causing the release of inflammatory factors such as IL-1β, IL-6 and TNF-α [26]. We found that the expression levels of inflammatory cytokines were significantly increased and cell-cycle procession was seriously inhibited following LPS-stimulation. Meanwhile, the expression level of *miR-322* was down-regulated and was opposite to the inflammatory cytokine mRNA level. Moreover, the anti-inflammatory expression level was up-regulated. The data revealed that *miR-322* may be involved in the regulation of LPS-mediated inflammatory response.

Surveying the predicted targets associated with inflammatory response of *miR-322*, one conserved 7-nt site in the NF-κB1 (p50) 3′-UTR is complementary to the *miR-322* ‘seed’ region. NF-κB is an important transcription factor that regulates the transcription and expression of multiple genes. It is closely related to the cell activation, cell proliferation, immune response and inflammatory reaction process [6]. The transcription factor p50, the transcript of NF-κB1, is an important subunit of NF-κB. It regulates gene transcription through combination with p65, which is another important subunit of NF-κB. When the signalling pathway is activated, p50/p65 exposes nuclear localization signal and translocates into the nucleus, regulating the transcription of inflammatory cytokine [2,6]. In the present study, the interaction between NF-κB1 (p50) and *miR-322* was analysed by luciferase assay. Luciferase expression level was significantly reduced when cotransfected with *miR-322* and WT-3′-UTR plasmid, whereas no significant modulation was observed with Mut-3′-UTR. Furthermore, we found that overexpression of *miR-322* reduced expression of NF-κB1 (p50) mRNA and protein. Meanwhile, the expression level of inflammatory cytokines was inhibited by *miR-322* following LPS-stimulation. LPS/TLR4/NF-κB signalling pathway plays a crucial role in cell inflammatory effect. *miR-322* can target 3′-UTR of NF-κB1 (p50), which indicated that TLR4 receptor and its signalling pathway may be involved in the process. Taken together, these results indicated that *miR-322* negatively regulated LPS-mediated inflammatory response by directly targeting NF-κB1 (p50).

NF-κB signalling cascade involved in macrophage inflammatory response. Macrophages initiate the innate immune response and deal with antigen to T-cells regulating the adaptive immunity [27]. Macrophages protect the body from infection and produce various inflammatory cytokines. Nevertheless, overproduced cytokines may cause cell damage and result in pathological condition. Previous studies reported that many miRNAs are useful modulators in maintaining cell homoeostasis by regulating cell-cycle proteins [28,29]. In the present study, the function of* miR-322* in cell proliferation and cell cycle was investigated in macrophages. We found that RAW264.7 cell cycle was arrested in G_0_/G_1_ phase and cell proliferation was significantly inhibited following LPS stimulation. We also found a decrease in *miR-322* expression level in LPS-treated RAW264.7 cells. It indicated that *miR-322* is involved in RAW264.7 cell proliferation. In the subsequent research, overexpression of *miR-322* increased cell cycle arrest at G_2_/M-phase and the number of cells in G_0_/G_1_ was significantly decreased. Besides, LPS-induced cell proliferation repression was rescued by *miR-322*. These data suggested that *miR-322* could promote cell proliferation and arrest cell cycle following LPS-stimulation.

In summary, we had proved for the first time that NF-κB1 (p50) was a novel target of *miR-322*. Our data provided evidence that LPS-induced down-regulation of *miR-322* and its subsequent effects on NF-κB-mediated transcriptional activity are responsible for fine-tuning inflammatory responses in RAW264.7 cells. Besides, *miR-322* plays a significant role in RAW264.7 cell proliferation. However, the specific mechanisms of *miR-322* regulation of cell-cycle procession and cell proliferation remain to be further elucidated. Overall, *miR-322* may provide a new perspective for the treatment of inflammatory diseases.

## Abbreviations

DMEM: Dulbecco's modified eagle medium
IL: interleukin
LPS: lipopolysaccharide
PRR: cells expressing pathogen-recognition receptor
qPCR: quantitative real-time PCR
SOSC1: suppressor of cytokine signalling 1
TAB: TAK1-binding protein
TLR: toll-like receptor
TNF: tumor necrosis factor
TRAF6: tumor necrosis factor receptor-associated factor 6
WT-3′-UTR: luciferase-wild-type NF-κB1–3′-UTR
